# Psychosocial work environment stressors for school staff during the COVID-19 pandemic: Barriers and facilitators for supporting wellbeing

**DOI:** 10.3389/fpubh.2023.1096240

**Published:** 2023-03-13

**Authors:** Liz R. Rolf, Liz Vestal, Ashley C. Moore, Nikole Lobb Dougherty, Nancy Mueller, Jason G. Newland

**Affiliations:** ^1^Brown School Evaluation Center, Washington University in St. Louis, Saint Louis, MO, United States; ^2^Office of the Provost, Washington University in St. Louis, Saint Louis, MO, United States; ^3^Division of Pediatric Infectious Diseases, Department of Pediatrics, Washington University School of Medicine, Saint Louis, MO, United States

**Keywords:** COVID-19, teacher retention, qualitative analysis, psychosocial work environment, pandemic stress, teacher burnout, psychological resilience, school environment

## Abstract

**Introduction:**

After periods of remote and/or hybrid learning as a result of the COVID-19 global pandemic, the return to in-person learning has been beneficial for both students and teachers, but it has not been without challenges. This study was designed to assess the impact of the return to in-person learning on the school experience, and efforts made to ease the transition in furthering a positive in-person learning environment.

**Materials and methods:**

We conducted a series of listening sessions with 4 stakeholder groups: students (*n* = 39), parents (*n* = 28), teachers/school staff (*n* = 41), and a combination of listening sessions and semi-structured interviews with building level and district administrators (*n* = 12), focusing on in-school experiences during the 2021–2022 school year amidst the COVID-19 pandemic. A primarily deductive qualitative analysis approach was employed to code the data followed by a primarily inductive thematic analysis, followed by thematic aggregation, thus providing depth and identification of nuances in the data.

**Results:**

Three main themes emerged around school staff experiences: (1) increased levels of stress and anxiety manifested in key ways, including students' challenges with personal behavior management contributing to increased aggression and staff shortages; (2) school staff described key contributors to stress and anxiety, including feeling excluded from decision making and challenges with clear and consistent communication; and (3) school staff described key facilitators that supported their management of anxiety and stress, including adaptability, heightened attention and resources to wellbeing, and leveraging interpersonal relationships.

**Discussion:**

School staff and students faced significant stress and anxiety during the 2021–2022 school year. Further exploration and identification of approaches to mitigate key contributors to increased stress and anxiety for school staff, along with increased opportunities for implementing key facilitators that were identified as important in managing and navigating the increased stress and anxiety offer valuable opportunities for helping to create a supportive work environment for school staff in the future.

## 1. Introduction

The coronavirus disease (COVID-19) pandemic significantly altered what it means to work in the field of education. As essential workers, school staff were on the frontlines of the virus and its impacts. Nearly overnight, they shifted their lesson plans and tools to accommodate virtual learning, often using platforms with which they and their students were unfamiliar; all while caring for themselves and their own household members during the early days of the pandemic in the United States. School staff have always played multiple roles beyond classroom management and lesson planning, serving as conduits for social-emotional development, family intervention and mediation, and connections to additional services for students and families.

In Missouri, all K-12 public schools (555 school districts and charter schools) closed for in-person instruction and activities on March 18^th^, 2020 (1, 2), for the remainder of the academic year in response to the pandemic, affecting almost one million students (3). However, such closures did not necessarily apply to teachers and other staff members, as some were still required to report to campus to provide academic instruction to their students who were learning remotely, as well as to support essential services like food distribution and childcare (3). School re-openings for the 2020–2021 school year varied by district in Missouri. St. Louis-area school districts varied widely, with some deciding on fully in-person instruction, some fully online, some hybrid (in-person and online), and some offering parents the choice between two or more of these models for students. Most school districts in the St. Louis area returned to a full schedule of in-person instruction for both students and staff by the by the fall semester of the 2021–2022 school year. Several Missouri schools were forced to abruptly close and temporarily return to virtual instruction due to a significant number of student absences and staffing shortages from COVID infections, exposures, and periods of quarantine, contributing to a sense of unpredictability in the school environment (4, 5). The 2020–2021 and 2021–2022 school years were marked by constant changes to methods of instruction and other rapid transitions. School leaders in Missouri and around the United States (U.S.) reacted in real-time to the best available data and recommendations as they worked tirelessly to keep the school community healthy while continuing to provide quality education (4, 5).

Teachers, students, and families experienced traumatic impacts due to the pandemic raising the likelihood of negative health outcomes, both psychological and physical (6). The return to in-person learning brought another period of transition for students and teachers. After becoming accustomed to often shortened school days while virtual, students had difficulty engaging in a full day of in-person instruction. Much of the preliminary research compiled by the U.S. Department of Education's Office of Civil Rights has shown the impact of the social-emotional and learning gaps displayed in students as they returned to in-person learning (6). As such, the mental health status of youth in the U.S. is currently recognized as a crisis and a national state of emergency was declared by the American Academy of Pediatrics (AAP), the American Academy of Child and Adolescent Psychiatry (AACAP), and the Children's Hospital Association (CHA) (7). Perhaps due to greater time spent at home as well as psychological and economic constraints, the number of gun-related homicides and suicides among youth increased after the pandemic and are nearly equivalent to the number of children who have died from COVID-19 (6). That equates to about one additional death per day as compared to pre-pandemic child mortality rates (8). In school, this resulted in negative impacts on students' behavior, classroom engagement, and social-emotional development. Teachers who work with such students have also reported experiencing emotional difficulties related to compassion fatigue or secondary trauma (9). In turn, teachers often bear the brunt of these impacts. This has further exacerbated the burden on teachers as they continue to have multiple responsibilities for the social, emotional, and academic success of their students.

Additionally, many COVID-19 leave policies in school districts differed from students to staff; due to their status as “essential workers,” school staff were often limited in their ability to take time off work to care for others in the home who were sick or recovering from COVID-19 (10). This burden led to increased levels of anxiety, stress, and burnout for school staff, including many leaving the teaching profession. The field of education has seen a mass exodus throughout the course of the pandemic; there was a net loss of ~600,000 educators working in public education in the United States from January 2020 to February 2022, per the U.S. Bureau of Labor Statistics (11). Since 2015, teacher retention rates have declined; through 2021, the average attrition rate for Missouri public school teachers was 11%, higher than the national average of 8% (12).

The pandemic only exacerbated pre-existing stressors for school staff, and highlighted the influence of teachers on their students (9). Adequate teacher support is necessary to mitigate job-related stress, which can be attributed in part to teacher shortages, weakened teacher mental health, and low-performing students (7). Many teachers reported feeling high levels of concern for their students' academic and emotional wellbeing, partly due to the increasing educational inequities exacerbated by the pandemic as well as a heightened sense of responsibility to meet their students' educational needs (9). Additionally, students' home life could have further contributed to teacher stress whereby trauma experienced at home while isolating during the pandemic may have contributed to increasing disruptive behaviors and declining academic performance among students (13).

Listening sessions discussed in this article were conducted as part of a study funded by the National Institutes for Health's initiative to support the Rapid Acceleration of Diagnostic Testing for Underserved Populations (RADx-UP), which focused on increasing access to COVID-19 testing for underserved and vulnerable populations (14). Researchers from Washington University in St. Louis partnered with five local school districts with predominately Black/African-American student populations to help increase access to testing as a strategy to reduce the spread of COVID-19. This Safe Return to Schools (SR2S) study sought to assess the best testing strategy to limit COVID-19 transmission in 16 St. Louis-area middle and high schools by providing frequent and free saliva-based COVID-19 testing through both weekly screening testing and symptomatic testing programs. Designed with a health equity lens, the SR2S study sought to decrease racial and health disparities related to COVID-19 among underserved and vulnerable populations, whose percentages of hospitalizations are higher than their population percentages (15).

One goal of the qualitative component of the study was to assess perceptions of the COVID-19 pandemic and the COVID-19 testing programs offered in these school districts at two different time points through listening sessions (focus groups) with students, parents, and school staff, and a combination of listening sessions and interviews with administrators. This article focuses on teachers' experience with returning to in-person school and shares findings from T2 to present considerations for fostering a supportive school environment.

## 2. Methods

A group of qualitative methodologists of the Safe Return to Schools (SR2S) research team conducted a series of listening sessions with students, parents/caregivers, and school staff at two timepoints to better understand their perspectives and experiences with COVID-19 testing, in-person school participation, and vaccinations. A combination of individual interviews and listening sessions were also conducted with building- and district- level administrators. Demographic information was collected from listening session participants. Data collection from time point 1 (T1) of the study took place from July to December of 2021 and examined perceptions around whether frequent testing would provide additional benefit, beyond current school strategies (i.e., distancing, masking, hand sanitizing, isolating) to prevent COVID infections. With the increased availability of COVID-19 vaccines, data collection for time point 2 (T2) occurred between April 6 and May 26 of 2022 and also included a focus on vaccine uptake with emphasis on understanding various facilitators and barriers to vaccination. The Washington University Institutional Review Board approved this study (IRB Approval #202104013).

### 2.1. Participants

School staff, school and district administrators, students, and parents/caregivers from five urban and suburban school districts in St. Louis, Missouri, were invited to participate in listening sessions. Listening session participants were recruited through distribution of flyers within various networks amongst community organizations and a community advisory board (CAB), which was comprised of students, parents/caregivers, school representatives (i.e., teachers, district leaders, nurses, school board members). Assembled to guide the design and implementation of the SR2S project, CAB members were selected for their proximate relationship to partnering school districts. Members participated in monthly virtual meetings and provided ongoing feedback on study activities such as reviewing project materials (e.g., listening session question guides, recruitment materials), helping to interpret and contextualize findings, and suggesting community resources to address study participants' needs and requests. Recruitment flyers were sent to school administrators and then sent electronically directly to parents/caregivers and staff through the schools existing communication modalities (e.g., *via* email, PeachJar—a school messaging application).

Participation in listening sessions was voluntary. Participants were given documents outlining the project overview and consent information. Verbal consent was provided at the beginning of each session for those who were 18 years and older. For students under 18, their parent/guardian electronically signed consent forms prior to the session. Participants were asked to provide their demographic information including gender, age, race, ethnicity, and highest level of education completed using a Qualtrics online tool. Additionally, participants were asked about their vaccination/booster status and if they had ever been tested for and/or tested positive for SARS-CoV-2. 70.83% (*n* = 85) of participants provided their demographics data (Table 1).

**Table 1:** Demographics of listening session participants.

	**School staff (*****n*** = **41)**		**Parent/caregivers (*****n*** = **28)**		**Students (*****n*** = **39)**	
	**Number**	**%**	**Number**	**%**	**Number**	**%**
**Gender**						
Male	4	9.8	0	0.0	8	20.5
Female	31	75.6	8	28.6	18	46.2
Non-binary	1	2.4	0	0.0	1	2.6
Missing	5	12.2	10	35.7	8	20.5
**Age**						
12–17	0	0.0	0	0.0	15	38.5
18–24	2	4.9	0	0.0	15	38.5
25–34	5	12.2	2	7.1	0	0.0
35–44	15	36.6	7	25	0	0.0
45–54	8	19.5	7	25	0	0.0
55–64	7	17.1	1	3.6	0	0.0
65+	0	0.0	1	3.6	0	0.0
Missing	4	9.8	10	35.7	9	23.1
**Race**						
White or Caucasian	21	51.2	2	7.1	11	28.2
Black or African American	12	29.3	15	53.6	18	46.2
Asian	1	2.4	0	0.0	1	2.6
Multiracial or Biracial	1	2.4	0	0.0	0	0.0
Prefer not to answer	0	0.0	1	3.6	0	0.0
Missing	6	14.6	10	35.7	9	23.1
**Ever tested for COVID-19**						
Yes	35	85.4	14	50.0	22	56.4
No	2	4.9	3	10.7	8	20.5
Missing	4	9.8	11	39.3	9	23.1
**Ever tested** ***positive*** **for COVID-19**						
Yes	12	29.3	5	17.9	2	5.1
No	22	53.7	10	35.7	18	46.2
Prefer not to answer	1	2.4	0	0.0	1	2.6
Missing	6	14.6	13	46.4	18	46.2
**WUSM testing**						
Yes (surveillance)	18	43.9	1	3.6	4	10.3
Yes (drive-up)	3	7.3	2	7.1	4	10.3
No	15	36.6	15	53.6	21	53.8
Unsure	1	2.4	0	0.0	0	0.0
Missing	4	9.8	10	35.7	10	25.6
**Vax status**						
Received all injections	35	85.4	13	46.4	13	33.3
Received some injections	0	0.0	1	3.6	5	12.8
Planning to get vaxxed	0	0.0	1	3.6	4	10.3
Not planning to get vaxxed	0	0.0	0	0.0	1	2.6
Not sure about getting vaxxed	1	2.4	1	3.6	5	12.8
Prefer not to answer	1	2.4	2	7.1	1	2.6
Missing	4	9.8	10	35.7	10	25.6

### 2.2. Strengths and limitations

Pre-existing partnerships, familiarity, and trust between members of the research study team and the relevant school communities significantly supported the recruitment of participants whose racial and ethnic demographics approximated that of the districts' demographics as a whole. The research team utilized a strengths-based approach (16–18) to the data collection. For example, the team examined the phrasing of questions for the listening session and interview guides for opportunities to ensure they were not deficit-based but rather strengths-based (e.g., “What are the top 2–3 things your school did well that helped you in returning to school in person?”). The team also applied an empowerment lens to the way in which demographic information in the survey was collected, and left these fields open-ended to allow participants to share ways they felt best captured how they see themselves (19–21). As a result of this approach, our participants had 15 unique responses to race, 27 unique responses to ethnicity, and six to gender. Our team distilled these categories in the following ways: Black or African American (included African, African American, Afro-American, American of African descent, Black African, Black American, Black Female, and Caribbean American); White or Caucasian (including White, Caucasian, German, Western European, and Jewish); Asian (including Korean and Indian American); and Multiracial or Biracial (including Western European/First Nation). Participants described their gender in the following ways: Female, Male, and Non-binary (including non-binary and AFAB but prefer they/them pronouns).

The demographics of the participants reflect an approximation of the districts' demographics as a whole. That being said, recruitment was limited to materials being distributed only within the existing school information-sharing structure and the school-associated COVID-19 testing program sites. This could cause us to have missed potential participants who are less engaged with or able to access these school resources, along with potential participants at schools where promotion of the study was less robust than other sites. Additionally, potential participants who deliberately did not engage with school-associated COVID-19 communications and resources, and who did not learn of the study through word-of-mouth or other informal means, could be underrepresented in the participant sample.

### 2.3. Instrumentation

Objectives for the listening sessions were as follows: to understand the perceived risks of COVID-19 for students and staff when on campus; to understand the social, behavioral, and ethical facilitators and barriers to testing, attending in-person school, and vaccination among parents/caregivers, students, and staff; to identify information and resources that are needed to keep students and staff in school; and to understand what role, if any, testing and vaccination play in mitigating perceived risks.

Participants were asked to provide feedback about the barriers and facilitators to participating in COVID-19 testing and vaccination, as well as their school- and district-level supports to reduce COVID-19 transmission. Facilitation guides were developed for each participant group (i.e., district/building administrators, school staff, students, and parents). The CAB and SR2S workgroup provided feedback to inform the final versions.

### 2.4. Procedure

We hosted a total of 21 listening sessions and interviews *via* Zoom with 120 participants–41 staff, 39 students, 28 parents/caregivers, and 12 administrators, lasting an average of 53 min (Table 2). Listening sessions and interviews were arranged by stakeholder group and took place virtually *via* Zoom (22). Text messages and two email reminders were sent prior to each session to those whom had signed up for a session. The team sent follow-up emails to no-show participants to offer future sessions for participation. Staff, students, and parents received a $50 electronic e-gift card for their participation. Administrators were not given an incentive for their participation. Listening sessions and interviews were recorded, with audio files sent to an external service for transcription. Transcripts were then formatted and edited in tandem with the audio files by research assistants.

**Table 2:** Listening sessions/interviews and participants.

	**Sessions (*n*)**	**Participants (*n*)**
**Interviews**		
District-level administrators	1	1
Building-level administrators	2	2
**Listening sessions**		
District-level administrators	1	4
Building-level administrators	2	5
Parents/caregivers	4	28
School staff	5	41
Students	6	39
Total	21	120

### 2.5. Data analysis

Leveraging an existing codebook from T1 that was modified for T2, the analysis team performed a directed thematic content analysis (23). This codebook was iteratively developed using a primarily deductive approach, drawing its content from the facilitation guides and research questions, resulting in 35 descriptive codes. From this point, the overall data analysis process was guided by the grounded-theory approach, where recurrent findings and themes primarily originate from the data itself through inductive analysis, as opposed to identifying data which relates to pre-identified themes and expected findings, such as would be utilized under deductive analysis (24–26). The grounded-theory approach was selected for its utility in identifying retrospective changes in participants' attitudes over time. Another factor for this consideration was grounded-theory's emphasis on building substantive theories from the gathered data, which are tailored toward more specific situations. In this way, results from a grounded-theory approach were more useful to informing policy and procedure development, as opposed to broader grand or formal theories which focus on addressing larger questions or concerns.

The 35 descriptive codes and the transcripts were imported into NVivo, a qualitative data analysis software, for testing of the codebook (27). Codes were applied to individual text units in the transcripts. As outlined for this study's process, each text unit consisted of the facilitator's question and the responses of participants. Where possible, individual participant responses to each question were formatted in the transcripts into their own text units to simplify analysis, so that individual responses to a question could be coded separately. Each text unit could be assigned as many codes as was appropriate for the content. To test the codebook, two team members (LV, AM) trained in qualitative analysis, independently coded the same transcript and then compared their coding. Three rounds of coding and comparison, with one transcript coded per round, occurred before reaching an acceptable level of validity and veracity of the codebook. After coding, inter-rater reliability (IRR), as measured by Cohen's kappa coefficient and the percentage of agreement between the coders for each code, were calculated for the test transcripts in NVivo (28). For any codes where the κ < 0.8 or the % agreement <85%, the coders met to discuss discrepancies in code application and reach a consensus on when to proceed. Coming to a consensus and resolving discrepancies on each round involved discussion between the coders of how they interpreted and applied the definition for any code whose individual IRR or percent agreement fell below the aforementioned thresholds, clarifying the definitions and inclusion/exclusion criteria for each such code, and coming to an agreement on their understanding of each code and how it should be applied. When the overall IRR reached the minimum of κ = 0.85 between the two coders' work, and the codebook was considered validated. The remaining 18 transcripts were split between the two team members and coded independently (29). All 35 descriptive codes could be applied to transcripts from any of the four stakeholder groups, although a few codes were more relevant to specific groups. Codes relating to questions on district-wide policies and procedures tended to be applied more frequently to transcripts from district-level administrators, for example. Upon completion of coding, no stakeholder group had at least one text unit coded to each of the 35 descriptive codes (administrators = 33, parents = 31, staff = 30, and students = 31).

From these coded transcripts, code reports were generated in and exported from NVivo. These code reports for each code were generated separately by stakeholder group and contained all text units which had been coded to an individual code. Each individual code report was then analyzed by a member of the study team (LR, AM, LV, NLD, AP, RB, RTB, and LN) to identify themes. Theme statements were created and accompanied with quotes which supported each theme, and grouped by code and stakeholder group. Theme reports were reviewed by a second team member and finalized through consensus between the writer and reviewers. A total of 532 initial theme statements across all stakeholder groups were identified (administrators = 143, parents = 135, staff = 131, and students = 123).

**Figure 1:**
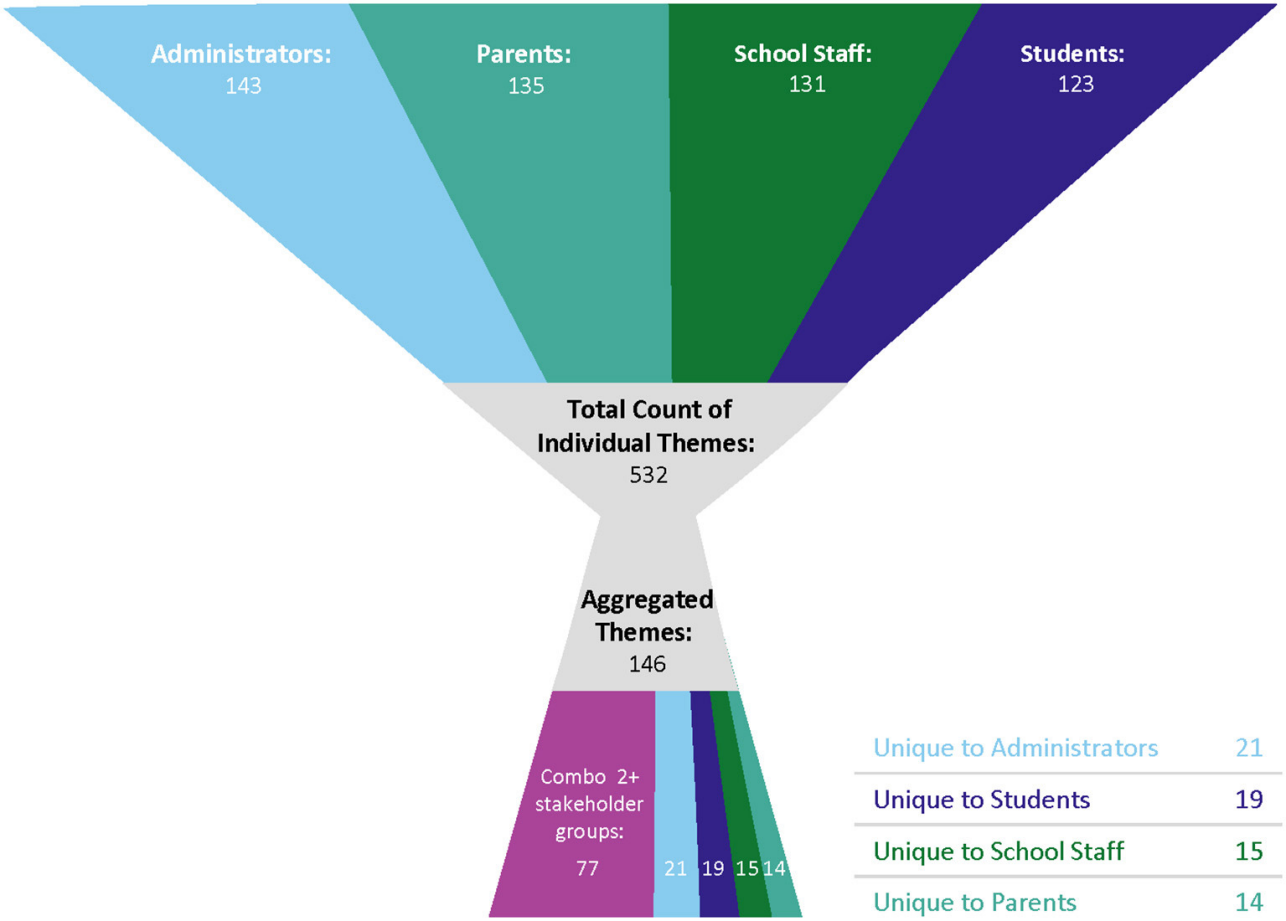
Theme statement count by stakeholder group.

The 532 theme statements resulting from the aforementioned directed thematic analysis were analyzed in an iterative thematic aggregation process, where the aim was to identify theme statements at each level by highlighting points of convergence and divergence, which resulted in 146 revised and aggregated theme statements. All theme statements were analyzed together to identify commonalities and repetition of themes within and between stakeholder groups and across theme domains (e.g., school experience, mitigation strategies). This began with analyzing all theme statements from a given stakeholder group, identifying very similar theme statements that were developed from evidence in different code reports, and combining and revising the very similar theme statements within each stakeholder group. From the theme statements, ten overarching domains of themes were identified across all stakeholder groups. After assigning a domain to every theme statement, team members (AM, LR, LV, NLD) further analyzed each theme statements to synthesize and refine theme statements across codes and stakeholder groups, as well as identifying uniqueness and/or convergence. Theme statements were then combined into revised aggregated/synthesized theme statements, resulting in the total of 146 aggregated theme statements across ten domains. Almost half of these aggregated theme statements were themes that were supported by evidence from two or more stakeholder groups. The presence of support for an aggregated theme statement by members of two or more stakeholder groups demonstrates intergroup consistency of these aggregated theme statements (Figure 1).

## 3. Results

The COVID-19 pandemic increased stress and anxiety for many. In the school environment, staff (administrators, teachers, and support staff) experienced unprecedented levels of stress and anxiety throughout the pandemic due in part to the frequently changing school environment (30–37). Throughout our listening sessions, three main themes emerged about the experience of school staff. First, re-adjusting to in-person learning increased levels of stress and anxiety, which manifested in two primary ways: challenges with personal behavior management and coping skills which contributed to an increase in students' negative behaviors (e.g., physical aggression) and staff shortages. Secondly, school staff described key contributors to increased stress and anxiety in their work environment, including feeling excluded from decision making in school policies and challenges with clear and consistent communication. Finally, school staff described key facilitators that supported their management of the increased anxiety and stress they were experiencing. These strategies included: fostering adaptability (e.g., learning to successfully teach concurrently in multiple modalities; building flexibility into lesson plans to accommodate quarantine periods); increased frequency and transparency of communication; heightened focus, attention and resources to socio-emotional learning and wellbeing; and building and leveraging trusting relationships both within and outside the school community.

### 3.1. Increased stress and anxiety experienced by school staff during readjustment to in-person learning manifested in key ways

Participants identified significant contributors to feelings of stress, anxiety, and burnout amongst staff members when returning to in-person learning.

#### 3.1.1. Challenges with personal behavior management and coping

Returning to in-person learning and instruction was a challenging transition for many. Upon the return to in-person learning, staff, students and parents/caregivers reported a significant decrease in personal behavior management and increases in physical aggression among students, particularly but not exclusively at the high school level. Examples of decreased student personal behavior management included excessive tardiness (“The amount of tardies that I mark every single day is just amazing. Or kids will just skip, and there's no remorse or apologies or anything like that. Kids are just, “It's fine. I don't really care.” Even when I reach out to parents,” per one teacher) and unapproved use of personal devices in class (“These cell phones to have become almost a norm. The Chromebooks, the kids can't seem to get off of it. They have really lacked that traditional learning and it's so hard for them to stay focused on what they're doing, because they're taking out their cell phones, they're texting each other, they're on the Chromebook, they want to listen to music, these little earbuds in their ears. That has been a big challenge.”). Of particular concern to many staff and parent participants was a marked increase in physical aggression and fights, which at times led to on-campus arrests of involved or suspected students. As one teacher described the violence, “We have seen a huge uptick in physical aggression in our school and it's been very out of character for our population in our district. And very violent fights, we've had kids end up arrested and it's almost as if they've forgotten, their stress levels are so high that they don't know how to interact with each other anymore. It tends to be very physically aggressive.” Staff and students alike felt stressed and emotionally challenged transitioning back to the in-person environment. For some staff, this was considered to be a powerful contributor to prompting themselves or their colleagues to leave or consider leaving the teaching profession. One teacher described their colleagues' experiences: “A lot of teachers I know are fed up, they're quitting and retiring, they had enough. We were dealing with behavior issues, more fights. I worked with kids as an educator for 20 years, I have never seen so many kids fight until this year. I don't know because of COVID, they stayed at home, what happened, they have no respect for teachers, no respect for other students. And I think it might be because of the pandemic.” Parents also shared their concerns about the more aggressive environment their students were exposed to (“[By the third week of school, my son saw] up to 37 fights. A couple of security guards up there, one time I went up there one security guard had his arm in a brace, the other one had his leg in a brace and that comes from them breaking up fights.”) and how the violence affected school staffing (“They say, because of all the fights, that [the schools] were short on staff.”).

#### 3.1.2. Staff shortages affect school environment

The staff shortage in St. Louis-area schools increased during the first two school years of the pandemic, as shown in early retirement and resignation rates and difficulties filling open positions (38). For participants we spoke with, school staff leaving the profession was perceived to have accelerated even further once students fully returned to in-person learning. The contributing factors to their departures were numerous, but many participants described an overwhelming sense of pressure and exhaustion, also referred to as burnout, as the primary factor. This sense of burnout was fueled by many different experiences and situations for individual participants, such as angry or aggressive behavior directed toward staff by both students and parents, and feeling that their schools supported them as best they could, but districts were unable to offer the extent of support they needed. This shortfall in staff support was noted by some parents, with one sharing that “all of [the school districts] are trying, but they're not supporting these teachers enough, the teachers shouldn't have to deal with kids fighting. […] We got to get things together. I mean, we really are worried about [curriculum content] and the teachers are walking in and going to the hospital, because they getting a book thrown at them or something, that is nuts.” One parent was particularly concerned about how the school bus driver shortage made it more difficult to comply with social distancing guidelines, as “it was just too many kids on one bus.”

As one would expect, staff shortages had a significant impact on the school environment, including student experience, prompting changes such as large class sizes, limited class offerings, increased deployment of inexperienced or long-term substitute teachers, and less access to individual support services (e.g., tutoring, afterschool activities). Partially due to the shortages, some school staff shared that they felt that they were unable to take paid time off (PTO), reducing their ability to rest and recover when needed. Staff shortages further increased burdens placed on colleagues who remained, and those we spoke with said this further impacted their ability or willingness to take PTO to care for themselves or family members. A teacher reflected on how staff shortages impacted other teachers, “Teacher staff is very short right now. Also, bus drivers, subs, we have none. We were told to come to work, try not to miss any days, basically sick and all because they had nobody to cover us which is ridiculous. I think the pandemic made teachers a dying career. A lot of people don't want to be teachers anymore. It's too stressful at this point.” One teacher shared their decision to leave the field after the 2021–2022 school year due to this, describing their experience and choice as “I'll just say the burnout. I can say personally, I'm leaving education after this year. I never anticipated that for myself, and I am beyond burnt out with just everything that we have dealt with over the past couple years. I want to say if I took more days, it would've been better, or if I would've done different things, it would've been better, but I don't know if that's true. It's just being able to recognize that. Obviously, I love my students and all of those things but those things take tolls on us.”

As a result of the pandemic, the school environment changed. The resources and supports school staff feel they need to feel healthy and well while at work evolved. As one teacher shared, “This is going to take a long time to recover… there's not a magic pill. There's not a magic formula or program. It's going to be a lot for not only our students, but for us as well. I think it's impacted me on not just a professional level but a personal level. And [if] all that veil between professional and personal went away during COVID and it's just changed the way that I view life now and what's important…where do we go from now? And trying to figure out what is the new path and the new normal because clawing at something that doesn't exist anymore is frustrating not to only our students, but I think to us as well. So, I think navigating that and finding the way forward is really hard and messy.”

### 3.2. Key contributors to increased stress and anxiety in the work environment

Participants identified key contributors that increased stress and anxiety in the school work environment, including staff members feeling excluded from decision making, and challenges with clear and consistent communication between schools, districts, and stakeholders.

#### 3.2.1. Feeling excluded from decision making and/or processes

Participants often discussed a desire for opportunities to be heard and included, whether in providing input on COVID-19 policies or feeling like a part of their school's community in general. When school staff felt excluded from being given chances to give input, it sometimes contributed to increased stress. For example, many participants, especially staff, felt excluded from involvement in contact tracing efforts. In particular, auxiliary staff, such as librarians and paraprofessionals, noted that they often found that they had been in contact with a student who had been quarantined within the contagious period through conversation with students and fellow staff, rather than being notified through their school's designated contact tracing protocol (per an auxiliary staff member, “They would follow the kids' schedules once they were positive to see where they were sitting, but they never checked in with the counseling office to see if I had met with anybody.”). Others noted inconsistencies in determinations of who might have been exposed by an infected student, such as in the example one staff member described where “one person could be quarantined, but then the person directly next to them wasn't, but maybe the person in front of them was.”

#### 3.2.2. Challenges with clear and consistent communication

Challenges with ability of clear and consistent communication increased stress and anxiety of many participants. School staff and administrators alike noted that keeping up with changing public health recommendations and communicating out any changes was difficult. In the words of one administrator, “And so, it was just the lack of clear and consistent messages was very challenging for all staff involved, and kids involved too because things were constantly changing. And yeah, I think it was just a hard toll on emotional wellbeing of staff. I mean, we've got a large number of staff that are leaving this year. And it's not a surprise, to be honest. It's been a tough year.”

A great deal of uncertainty was encountered by all throughout the pandemic. Many school staff experienced increased communication (through meetings, emails, etc.) from administrators, colleagues, and parents during this time. Increased communication provided comfort and connection and helped to reduce anxiety of some. Staff felt particular stress around the lack of communication when they had a potential exposure to COVID-19. One staff member commented, “We just didn't really find a rhythm for a while about being able to communicate... But also [COVID-19 status] being confidential, not really wanting to reveal who was the exposure, [where] exposure came from.”

Some administrators discussed that their biggest lesson learned throughout the pandemic was the importance of clear, frequent communication with students and families using a variety of modalities, allowing families to choose how they wanted to engage. One example where this was made evident was in contact which included formal methods (e.g., town halls, surveys, weekly emails or videos) and informal methods (e.g., making themselves available daily for in-person interactions with the school community during drop-off and pick-up times). As one administrator recommended, “Hindsight's always 20/20, but you make the best decisions you can based on the information you have, and try to be as open and honest with your community as possible, and provide the information you have, and what you know. And people seem to understand that, respect that if they think you're just being honest.”

### 3.3. Key facilitators that supported navigating increased anxiety and stress of school staff

While acknowledging the complexity and messiness of the challenges of fostering a positive work environment, participants mentioned multiple examples of strategies and supports that aided them in navigating the stressful school environment during the pandemic. These approaches including fostering an adaptable mindset and work environment, heightening attention to and resources for socio-emotional learning and wellbeing, and building and leveraging trusting relationship both within and outside the school community.

#### 3.3.1. Fostering adaptable mindsets and work environment

School environments were continually adapting amidst constant change and uncertainty throughout the pandemic. All stakeholder groups emphasized the importance of allowing for and fostering flexibility through policies and practices, especially those that aimed to facilitate the wellbeing and mental health of the entire school community. Throughout the pandemic, changes to public health guidelines and school-level recommendations were perceived by participants to become more frequent, particularly over fall 2021 and into the beginning of winter 2022. This made it exceedingly difficult for districts/schools to develop and implement consistent protocols. For school staff, this often required them to become adaptable and flexible in their ability to pivot between planned coursework and differing student attendance modes. As described by one teacher, “I might have all my kids on Monday, but on Wednesday I might have three or four kids in that class that are quarantined. Or by the end of the week, I might have five kids in a class that's quarantined. Now they all have different dates that they can return, so I had to learn to be flexible.” Having experienced so much uncertainty and change within their schools, many participants shared that an important lesson they learned is how to adapt their minds, behaviors, and schedules to the unpredictable, particularly their schools' ever-changing pandemic policies (i.e., virtual learning, masking, social distancing, quarantining, sanitizing). One staff member described their adaptability as such: “At first there [was] masks or no mask or shots or no shots, or some people were sick. Sometimes we needed to shut down. Sometimes we needed to clean. You just got to be open to the different things because everybody is still learning.” Some students also learned to be more flexible and responsive to changes in school policies and mitigation guidelines, with one noting “The biggest thing that I have learned is adaptation and balance just within myself and also within the school, because the guidelines are constantly changing. Seems like almost every other month we have a new variant. So as for the school, they're trying to juggle all of this. We have a lot of schools within the district, so they're trying to juggle it and accommodate everyone, keep students and families informed.”

#### 3.3.2. Heightened attention and resources given to social emotional learning, sense of security, and wellbeing

School staff shared that to alleviate some of the stress and anxiety felt within the school environment, it was helpful to see increased focus, attention, and resources given to socio-emotional learning and wellbeing. Some administrators worked within their schools to provide mental health resources specifically to teachers, such as counseling referrals, reading materials, and space to talk with their peers. In the words of one administrator: “So go easy on yourself, give yourself a pat on the back because students are not the only ones stressed out, staff is stressed out as well. So not just providing, those services for our students, but having professionals available to support staff as well. I think that helps because if a staff member is stressed out or traumatized, we know what that does to a student who's already traumatized. So I think one of the big takeaways is providing those supports for our staff so that they are being well in order to do well.” This contributed to staff prioritizing and focusing on their own mental health needs and outlets, setting boundaries, and being empathetic toward themselves and others, which aided in developing their psychological resiliency As described by a staff member: “Mental health is everything, and if you need to take some time away you need to take that time and not feel bad about it and realize that if you're not healthy, you're not going to be able to do the job that's put before you.”

In the school environment, the attention and care of strong student-teacher relationships in supporting students' academic and socioemotional growth was particularly prominent for all stakeholder groups. “This year has been bar none one of the best years of my teaching career. I would say just because the students kind of had an emotional growth mindset. They were willing to create, and latch onto relationships with me and with each other, and because their openness to build those relationships, […] I've never had a year like this in my 18 years of teaching. In that respect, they were so hungry for relationships, and they were so open it was magical.” Some teachers discussed the time and effort they spent incorporating socioemotional lessons, activities, and connections to resources into both their planned courses and informal conversations with students. One teacher encouraged empathy as a positive coping skill: “I think for me bringing that social, emotional learning piece into the classroom with the kids has been one of the things teaching them to have empathy. That's one of things that we really focused on teaching the kids to deal with their emotions, because believe it or not it has affected our kids greatly.” These efforts were discussed as endeavors that staff determined were worthwhile to integrate into their lesson plans, independent of district instructions. Teachers, staff, and administrators alike noted how important it was to extend grace, empathy, and compassion to their students and colleagues, acknowledging the impact and trauma the pandemic had on staff. Students responded in kind, and some recognized and appreciated how much value they now placed on getting to know their teachers, and how their teachers were working to support them in re-adjusting to the return to the in-person environment. As one student said of their school environment, “I understand the world is different right now, considering the current situation of the virus and everything, so we just have to look out for one another and take care of [each other].” Some administrators even incorporated efforts targeting general health and wellness in plans for school staff, such as offering Zumba classes to help relieve stress and support physical health. These efforts were considered valuable by our participants across stakeholder groups, some of whom noted that they were in response to the difficulties some students faced in re-adjusting to in-person learning and the school environment. Students noted and appreciated these efforts by school staff and administrators, as one student described: “They understand that it will take time for us to adapt back to the physical class system. So they try to move along with us and help us adapt back to the physical class system.”

Physical resources—whether cleaning and sanitation wipes, signage to encourage social distancing, or other supplies—were seen by school staff as an indication of school- and district-level leadership responding to their staff's wellbeing needs. One administrator noted that resources leveraged within various procedures to promote social distancing, hallway spacing, and accessible sanitation supplies helped meet staff members' needs to improve their sense of security and stability on campus: “And so by taking time and really spending a lot of time developing and then teaching, and practicing all of those procedures that we put in place, it assisted people with allaying their fears because in my experience, when you have systems in place and people know that the systems are going to be reliable and they learn to trust those systems, it can create a level of comfort for everyone.” As students began returning to in-person learning either fully or partially during the 2020–2021 school year, sanitation supplies (e.g., Lysol wipes, hand sanitizer stations) were abundant and enforcement of mitigation measures (e.g., masking and social distancing) was noted as strict in many schools. Many school staff perceived these resources as facilitators to increasing their comfort with being in-person at school. As one teacher noted, “I just feel like they were trying to give us everything they could possibly give us to try to help ease our worries.” Many participants across all stakeholder groups shared that these practices had a positive impact on morale and comfort levels for staff (particularly teachers), students, and parents in returning to campus. Administrators described their commitment to ensuring staff had the physical resources they needed to feel safe and secure. These efforts were noted and appreciated by staff, students, and parents.

#### 3.3.3. Building and leveraging trusting relationships both within and outside the school community

Efforts to support positive mental health and reduce stress and anxiety among school staff often focused on the human connections found within a healthy school community. As one school administrator stated, “…just the human connection. I think we took for granted the importance of being in community in school… school is about community. And the core of what we do was stripped away and forced to happen over a screen. And that was just, in my opinion, just devastating for our families and students.” For those we spoke with, the time spent in virtual learning highlighted the importance and value of interpersonal relationships and human interaction. As one teacher shared, “The biggest thing is to always remember is that, I don't want to say this in a super cheesy way, but just never forget the people, the actual students that they're there, to connect with them on a personal level. Because it's like just remembering to hold onto that, the humanity of it, the connection was something that just to cherish that.” For one school, an administrator focused on communication, human connection, and wellness in their efforts: “But also we did a lot of what I consider reading and just studying just how everyone else was feeling. Because human nature thinks that I'm the only one going through this. But as we were engaging teachers around, all teachers are feeling this way. So we were bringing in articles, we were bringing in certain books, we were having certain discussions. And I think that supported some of our mental and... I'm not going to say physical, but a lot of our mental. And we did a lot of, I call it community. We'd get lunch and we'd have staff meeting with all, or we bring doughnuts in the morning. So we worked on what's called community.” These efforts were noted and appreciated by some staff members and some shared a desire to see such offerings sustained or even increased in the future.

Building close interpersonal relationships within the school community helped many navigate and/or alleviate stress and anxiety of the ever-evolving school environment. Equally important became the building and leveraging of interpersonal relationships with others traditionally seen as outside the school environment, such as medical professionals and public health officials in the region. School administrators around the country were put in the position to make difficult decisions about school openings and closings, policy changes, and mitigation strategies throughout the pandemic. The vast majority of administrators with whom we spoke did not have public health expertise, but valued data-driven decision making, and so turned to local and national experts to inform their decision-making processes. School and district administrators made a point of ensuring that their schools' mitigation measures, particularly their quarantine protocols, complied with CDC guidance, and that such protocols were updated as the guidance changed. In the words of one school administrator regarding the district administrator in charge of mitigation policies for the district, “He wasn't making this stuff up as we went along He was real in touch with [local physician], the CDC, medical professionals…He used people who were experts at this, and he used their expertise to guide his decision. We were working from a place of science.” Staff, parents, and students were often appreciative of the fact that their schools' protocols were based on expert consensus, and adapted according to published research and recommendations.

In addition to collaborating with public health and medical experts, school administrators often connected with other administrators in the region to get feedback and share their plans. The difficulties in developing these plans were most evident for all stakeholder groups in regards to contact tracing and quarantine protocols, which were often perceived as inconsistent and at times contradictory. At the administration level, one administrator shared, “I serve on the secondary association of school principals for the region and there's 10 of us on this board from all different schools and all different districts and the commonality that because this was all going on, you're building the airplane in the air 10,000 feet and you're writing the draft on a cocktail napkin. We would reach out to each other regularly and be like, what are you doing over here? What are you doing over there?” The connections made across districts administrators during the pandemic helped them to feel reassured and connected.

## 4. Discussion

The goal for this supporting aim of the SR2S study was to better understand the perceptions of COVID-19 testing and vaccination across the four stakeholder groups in these districts, the impact of virtual learning, and the thoughts and experiences of stakeholders during the return to in-person learning. As a broad investigation, our findings cut across many different potential avenues for implementation and dissemination. Here we focus on factors contributing to the stress and anxiety experienced by school staff, the structural supports and resources that either contributed to or hindered a positive work environment, and the creative and flexible approaches they have taken to help manage that same stress and anxiety in their classrooms, as viewed through the lens of all four stakeholder groups. Our data suggest that the school environment, regardless of district, is a highly demanding workplace for teachers and school staff. This environment is the result of both long-term challenges in the teaching profession and the sudden, dramatic paradigm shift imposed on education by the COVID-19 pandemic. These findings on the mental health and wellbeing of school teachers and staff complement recent findings in research conducted to assess changes in mental health and wellbeing of students of all ages, due in part to the altered school and social dynamics imposed by pandemic-era adaptations. Both stakeholder groups have demonstrated increases in reports of anxious and depressive symptoms and related diagnoses (39–46). The pandemic brought many of the issues facing school staff into the broader public discourse and pushed them past the “tipping point,” so to speak, prompting both a mass exodus of teachers and a heightened emphasis on the socioemotional health and needs of both students and teachers, as provided within the school environment. Our findings offer insights into some of the factors fueling growing teacher resignation rates in the field, as prompted or worsened by the pandemic. In parallel, these findings also highlight some districts' practices and provide some considerations for efforts to address the socioemotional stressors and burdens resulting from the pandemic, particularly efforts to offer socioemotional health and wellbeing supports and resources to students and staff.

First, as has been widely covered in the public discourse, the pandemic has been a source of stress and anxiety for the majority of the population. School staff and students face a more demanding campus environment than they did prior to the pandemic, fueled in part by challenges students experience with re-integrating into the in-person learning environment, after spending many months in a virtual learning environment with far less human interaction. Aggression and negative behaviors increased for students, which was considered a significant contributing factor to increased teacher resignation rates in the participating districts. As the number of teachers in a district declines, the burden placed on the remaining teachers intensifies, further increasing the stress and anxiety many experience (47). Helping provide strategies and supports for students to cope with challenges in and out of school will allow them to have a strong foundation for re-adjusting to campus life. When on campus, incorporating socioemotional learning and coping strategies into existing lesson plans can also help students learn to better manage their emotions and contribute to a calmer school environment for all.

Second, sustained and increased communication within school communities would contribute to the feeling of a safe and supportive work environment for school staff (48). Communications should foster transparency as policies change, or the ability to provide feedback when policy changes occur, so that school staff feel included in an ongoing dialogue. Even when things do not change, several staff referenced frequent and transparent communication as a facilitator to feeling supported and heard by their school or administrators.

Third, building a supportive campus environment for both students and staff has the potential to significantly reduce the stress and anxiety teachers experience, thus supporting teacher retention and student success (49, 50). For essential workers, particularly those working in smaller spaces with large populations, understanding the physical resources that help employees feel safe and comfortable in their work environment (e.g., hand sanitizer, cleaning wipes, and masks) may be an important step.

Lastly, there are efforts underway to provide behavioral health resources and supports to students and staff, encourage stronger student-teacher relationships, bolster socioemotional learning in class lessons, and build a more trusting, compassionate, and empathetic school environment. Our research suggests that expanding these efforts would be supported by and valuable to students, staff, and parents in the school districts. Some participants discussed these efforts as one overall strategy to address the increase in students' negative behaviors within the schools and improve the work environment for school staff.

Moving forward, further research on this topic could explore a multitude of different questions and paths. Based on our findings, stakeholders in the school community, particularly teachers, would value research aimed at identifying effective means of addressing and reducing the heightened rate of aggressive behaviors in schools, approaches that increase clear and consistent communication and expand opportunities to garner input from a broader set of stakeholders, and approaches for building and leveraging trusting relationships within and outside the school community. Throughout all of the socioemotional burdens teachers have carried during this pandemic, as both educators and individuals, there remains the sense that, despite the difficulties wrought by the return to campus, some things are getting better. The knowledge gained and next steps for future research aim to build on that sentiment. In the words of one long-time teacher, “Even though this year was still difficult, I actually felt momentum, unlike last year, which felt like doggy paddling on the best days. Consistency, relationships, in-person expectations, and teamwork, those are so crucial to school success.”

## Data availability statement

The datasets presented in this article are not readily available because this is qualitative data, and as such, any potential data sharing must first be approved by our IRB. Requests to access the datasets should be directed to LR, rolfl@wustl.edu.

## Ethics statement

The studies involving human participants were reviewed and approved by Institutional Review Board, Washington University in St. Louis. Written informed consent to participate in this study was provided by the participants' legal guardian/next of kin.

## Author contributions

JN was the primary contributor to conceptualization of the work. JN, NL, and NM contributed to the conceptualization and design of the study aim. LR, LV, AM, and NL contributed to data acquisition and initial analysis and conducted the qualitative thematic analysis and aggregation. LR, LV, AM, NL, NM, and JN contributed to manuscript content. LV and AM wrote the first draft of the Introduction section. LR wrote the first drafts of the Abstract, Methods, Results, and Discussion sections. LR, LV, and NL provided revisions to content, manuscript structure, and framing. JN and NM provided revisions to manuscript. LV developed the included tables and figure, with support from AM. AM managed and formatted the citations. LV formatted the manuscript. All authors contributed to manuscript revisions and read and approved the submitted version.

## References

[1] Ballotpedia. School Responses in Missouri to the Coronavirus (COVID-19) Pandemic. Middleton: Ballotpedia (2022). Available online at: https://ballotpedia.org/School_responses_in_Missouri_to_the_coronavirus_(COVID-19)_pandemic (accessed November 8, 2022).

[2] Clancy S. List of Major School Districts Closed in the St. Louis Area Due to Coronavirus Concerns. (2020). 10.1063/PT.6.2.20200302a

[3] Huguelet A. All Missouri Public Schools Temporarily Closed, Governor Parsons Says. New York, NY: Springfield News-Leader (2020).

[4] Camera L. Virtual Learning, Staff Shortages, Omicron Cases Rise in Big-City Schools. New York, NY: US News & World Report (2022).

[5] Bernhard B. Missouri School Districts Struggle to Keep Classrooms Open During COVID-19 Surge. St Louis: St Louis Post-Dispatch (2022).

[6] Department of Education. Education in a Pandemic: The Disparate Impacts of COVID-19 on America's Students. Washington, DC: Department of Education: Office of Civil Rights (2021). Available online at: https://www2.ed.gov/about/offices/list/ocr/docs/20210608-impacts-of-covid19.pdf (accessed November 8, 2022).

[7] American Academy of Pediatrics. AAP-AACAP-CHA Declaration of National Emergency in Child and Adolescent Mental Health. Washington, DC: American Academy of Pediatrics (2021). Available online at: https://www.aap.org/en/advocacy/child-and-adolescent-healthy-mental-development/aap-aacap-cha-declaration-of-a-national-emergency-in-child-and-adolescent-mental-health/?_ga=2.162909884.182485643.1666035029-1838847977.1662561727 (accessed November 8, 2022).

[8] Peña PA Jena A. Child deaths by gun violence in the US during the COVID-19 pandemic. JAMA Netw Open. (2022) 5:e2225339. 10.1001/jamanetworkopen.2022.25339 35925607 PMC9353595

[9] Robinson LE Valido A Drescher A Woolweaver AB Espelage DL LoMurray S. Teachers, stress, and the COVID-19 pandemic: a qualitative analysis. School Ment Health. (2022) 2022:1–12. 10.1007/s12310-022-09533-2 35875185 PMC9288205

[10] Moxley E. In Some Missouri School Districts, Teachers are Essential Workers: So They Don't Have to Quarantine. Missouri: NPR in Kansas City (2020).

[11] Walker T. Survey: Alarming Number of Educators May Soon Leave the Profession. Washington, DC: NEAToday (2022).

[12] Missouri Department of Elementary Secondary Education. DESE Launches Statewide Partnership to Tackle Missouri's Teacher Shortages. Missouri: Missouri Department of Elementary and Secondary Education (2021). Available online at: https://dese.mo.gov/communications/news-releases/DESE%20Launches%20Statewide%20Partnership%20to%20Tackle%20Missouri%E2%80%99s%20Teacher%20Shortages (accessed November 8, 2022).

[13] Cenat JM Dalexis RD. The complex trauma spectrum during the COVID-19 pandemic: a threat for children and adolescents' physical and mental health. Psychiatry Res. (2020) 293:113473. 10.1016/j.psychres.2020.113473 33198045 PMC7534660

[14] RADx-UP. RADx-UP. (2022). Available online at: https://radx-up.org/ (accessed November 8, 2022).

[15] Centers for Disease Control Prevention. COVID-19 Case Surveillance Public Use Data. New York, NY: Centers for Disease Control and Prevention (2022). Available online at: https://data.cdc.gov/Case-Surveillance/COVID-19-Case-Surveillance-Public-Use-Data/vbim-akqf (accessed November 8, 2022).

[16] Butler-Barnes ST Leath S Williams A Byrd C Carter R Chavous TM. Promoting resilience among African American girls: racial identity as a protective factor. Child Dev. (2018) 89:e552–71. 10.1111/cdev.12995 29154406

[17] Milner HR. Race, culture, and researcher positionality: working through dangers seen, unseen, and unforeseen. Educ Res. (2007) 36:388–400. 10.3102/0013189X07309471

[18] Cole ER. Intersectionality and research in psychology. Am Psychol. (2009) 64:170–80. 10.1037/a0014564 19348518

[19] O'Neil C. Weapons of Math Destruction: How Big Data Increases Inequality and Threatens Democracy. New York, NY: Crown (2017). p. 290.

[20] Fernandez T Godwin A Doyle J Verdin D Boone H Kirn A. More comprehensive and inclusive approaches to demographic data collection. In: Proceedings of the 2016 ASEE Annual Conference and Exposition Proceedings. New Orleans, Louisiana: ASEE Conferences (2016). p. 25751. Available online at: http://peer.asee.org/25751 (accessed November 9, 2022).

[21] Helms JE Talleyrand RM. Race is not ethnicity. Am Psychol. (1997) 52:1246–7. 10.1037/0003-066X.52.11.1246

[22] Zoom. Security Guide. San Jose: Zoom video communications (2021). Available online at: https://explore.zoom.us/docs/doc/Zoom-Security-White-Paper.pdf (accessed November 9, 2022).

[23] Hsieh HF Shannon SE. Three approaches to qualitative content analysis. Qual Health Res. (2005) 15:1277–88. 10.1177/1049732305276687 16204405

[24] Chapman A Hadfield M Chapman C. Qualitative research in healthcare: an introduction to grounded theory using thematic analysis. J R Coll Phys Edinb. (2015) 45:201–5. 10.4997/jrcpe.2015.305 26517098

[25] Chun Tie Y Birks M Francis K. Grounded theory research: a design framework for novice researchers. SAGE Open Med. (2019) 7:205031211882292. 10.1177/2050312118822927 30637106 PMC6318722

[26] Corbin JM Strauss AL. Basics of Qualitative Research: Techniques and Procedures for Developing Grounded Theory. 4th edn. Los Angeles: Sage (2015). p. 431.

[27] NVivo. Best Qualitative Data Analysis Software for Researchers. Singapore: NVivo (2022). Available online at: https://www.qsrinternational.com/nvivo-qualitative-data-analysis-software/home (accessed November 7, 2022).

[28] Cohen J A. Coefficient of agreement for nominal scales. Educ Psychol Meas. (1960) 20:37–46. 10.1177/001316446002000104

[29] Bradley EH Curry LA Devers KJ. Qualitative data analysis for health services research: developing taxonomy, themes, and theory. Health Serv Res. (2007) 42:1758–72. 10.1111/j.1475-6773.2006.00684.x 17286625 PMC1955280

[30] Sokal LJ Trudel LGE Babb JC. Supporting teachers in times of change: the job demands: resources model and teacher burnout during the COVID-19 pandemic. IJCE. (2020) 3:67. 10.11114/ijce.v3i2.4931

[31] Weißenfels M Klopp E Perels F. Changes in teacher burnout and self-efficacy during the COVID-19 pandemic: interrelations and e-learning variables related to change. Front Educ. (2022) 6:736992. 10.3389/feduc.2021.736992

[32] Rǎducu CM Stǎnculescu E. Teachers' burnout risk during the COVID-19 pandemic: relationships with socio-contextual stress—a latent profile analysis. Front Psychiatry. (2022) 13:870098. 10.3389/fpsyt.2022.870098 35546926 PMC9082493

[33] Westphal A Kalinowski E Hoferichter CJ Vock M. K−12 teachers' stress and burnout during the COVID-19 pandemic: a systematic review. Front Psychol.(2022) 13:920326. 10.3389/fpsyg.2022.920326 36118449 PMC9479001

[34] Shimony O Malin Y Fogel-Grinvald H Gumpel TP Nahum M. Understanding the factors affecting teachers' burnout during the COVID-19 pandemic: a cross-sectional study. PLoS ONE. (2022) 17:e0279383. 10.1371/journal.pone.0279383 36584003 PMC9803224

[35] Kotowski SE Davis KG Barratt CL. Teachers feeling the burden of COVID-19: impact on wellbeing, stress, and burnout. Work. (2022) 71:407–15. 10.3233/WOR-210994 35068412

[36] Gillani A Dierst-Davies R Lee S Robin L Li J Glover-Kudon R. Teachers' dissatisfaction during the COVID-19 pandemic: factors contributing to a desire to leave the profession. Front Psychol. (2022) 13:940718. 10.3389/fpsyg.2022.940718 36186287 PMC9518793

[37] Metrailer GM Clark KN. Returning to school: teachers' occupational and COVID-19-related stress and their perceptions of school climate. Contemp School Psychol. (2022) 2022:1–13. 10.1007/s40688-022-00428-2 36259075 PMC9559161

[38] Missouri Department of Elementary Secondary Education. Recruitment and Retention Report | Missouri Department of Elementary and Secondary Education. Available from: https://dese.mo.gov/media/pdf/recruitment-and-retention-report (accessed November 7, 2022).

[39] Hou J Yu Q Lan X. COVID-19 infection risk and depressive symptoms among young adults during quarantine: the moderating role of grit and social support. Front Psychol. (2021) 11:577942. 10.3389/fpsyg.2020.577942 33488448 PMC7820677

[40] Dumitrache L Stănculescu E Nae M Dumbrăveanu D Simion G Talo? AM. Post-lockdown effects on students' mental health in Romania: Perceived stress, missing daily social interactions, and boredom proneness. Int J Environ Res Public Health. (2021) 18:8599. 10.3390/ijerph18168599 34444348 PMC8394079

[41] Holm-Hadulla RM Klimov M Juche T Möltner A Herpertz SC. Wellbeing and mental health of students during the COVID-19 pandemic. Psychopathology. (2021) 54:291–7. 10.1159/000519366 34564075 PMC8678268

[42] Takács J Katona ZB Ihász F. A large sample cross-sectional study on mental health challenges among adolescents and young adults during the COVID-19 pandemic at-risk group for loneliness and hopelessness during the COVID-19 pandemic. J Affect Disord. (2023) 325:770–7. 10.1016/j.jad.2023.01.067 36681303 PMC9847220

[43] Pepe A Farina E. A mixed-method study on adolescents' wellbeing during the COVID-19 syndemic emergency. Sci Rep. (2023) 13:871. 10.1038/s41598-022-24007-w 36650194 PMC9843112

[44] Heck CJ Theodore DA Sovic B Austin E Yang C Rotbert J. Correlates of psychological distress among undergraduate women engaged in remote learning through a New York City college during the COVID-19 pandemic. J Am Coll Health. (2023) 47:1–10. 10.1080/07448481.2022.2156797 36649543 PMC10350472

[45] Kwaning K Ullah A Biely C Jackson N Dosanjh KK Galvez A. Adolescent feelings on COVID-19 distance learning support: associations with mental health, social-emotional health, substance use, and delinquency. J Adolescent Health. (2023) 12:5. 10.1016/j.jadohealth.2022.12.005 36653259 PMC9870620

[46] Xu T Wang H. High prevalence of anxiety, depression, and stress among remote learning students during the COVID-19 pandemic: evidence from a meta-analysis. Front Psychol. (2023) 13:1103925. 10.3389/fpsyg.2022.1103925 36704682 PMC9871576

[47] Vaillancourt T Brittain H Krygsman A Farrell AH Landon S Pepler D. School bullying before and during COVID-19: results from a population-based randomized design. Aggress Behav. (2021) 47:557–69. 10.1002/ab.21986 34235773

[48] Weiner J Francois C Stone-Johnson C Childs J. Keep safe, keep learning: principals' role in creating psychological safety and organizational learning during the COVID-19 pandemic. Front Educ. (2021) 5:483. 10.3389/feduc.2020.618483

[49] Toropova A Myrberg E Johansson S. Teacher job satisfaction: the importance of school working conditions and teacher characteristics. Educ Rev. (2021) 73:71–97. 10.1080/00131911.2019.1705247

[50] Johnson SM Kraft MA Papay JP. How context matters in high-need schools: the effects of teachers' working conditions on their professional satisfaction and their students' achievement. Teach Coll Rec. (2012) 114:1–39. 10.1177/01614681121140100427409075

