# Supplementary material for: Psychosocial work environment stressors for school staff during the COVID-19 pandemic: Barriers and facilitators for supporting wellbeing
Source: Front Public Health. 2023 Mar 13;11:1096240. doi: 10.3389/fpubh.2023.1096240 (PMC10040557; doi:10.3389/fpubh.2023.1096240)
Supplement: Supplementary file 1 [file Data_Sheet_1.DOCX]

**T2 Listening Session Guide: Building Administrators**

| 1. Please tell us your name, position, school, and district as well as how many years you’ve worked as a school administrator. **[Note: All participants must respond to this question]** |
| --- |
| ***Let’s start with the successes, challenges, and lessons learned that you and your school have experienced related to COVID-19 this past school year.***   1. Overall, what 2-3 strategies and practices do you think were critical to have in place this school year? How did these strategies and practices change over time? How were they different from last school year?    1. *Probes: policies, processes, mitigation strategies -- offering choice of virtual v. at school, classroom pods, PPE for staff and students, closing common areas/shared spaces…* |
| 1. More specifically, identify 2 or 3 things that have worked well in supporting your **teachers and** staff with in-person learning.    1. *Probes: PD, vacation, pay, childcare…* 2. What challenges did your **teachers and staff** experience with in-person learning? And how were you able to address these challenges?    1. *Probes: teacher shortages, sick leaves, filling in for teachers, cleaning classrooms…* 3. What about **students and families**? What are the top 2 or 3 things that worked well to support them with in-person learning?    1. *Probes: hybrid scheduling, remote learning, school meals, childcare, laptops/tablets, PPE’s…*      1. What challenges have **students and families** experienced related to COVID this school year? And how did you address these challenges?    1. *Probes: financial, transportation, communication, health, COVID testing, PPE’s, academic, behavioral/emotional, language barriers, technology, attendance…* 2. Which resources or information do you believe are necessary to keep students and staff at school? 3. From your experience, how easy or hard was it for students to succeed when returning to in-person learning? In light of the pandemic, what factors (in and out of school) play a key role in student success or failure? 4. With the emergence of the Omicron variant at the end of winter break, what factors or reasons influenced how your staff and families chose to respond to this variant or COVID in general?    1. *Probe: any social, behavioral, financial, or ethical factors (cultural norms, politics, health, income, community values)…*    2. Was it easy or hard to collect this information from staff and families? If easy, why? If hard, what communication barriers did you encounter? 5. Thinking about all that you have just shared, how has your role and responsibilities as a principal or assistant principal changed as a result of the pandemic? |
| ***Now let's move on and discuss testing and vaccinations.***   1. What risks related to COVID-19 occurred with in-person learning? How have those risks changed over this semester and within the past year? 2. How important was COVID-19 testing for your school community? 3. How easy or difficult was COVID-19 testing for your school community?    1. *Probe: Were there any challenges to access and for whom?* 4. What role do vaccinations currently play in your school community and district? In the future?    1. *Probe: Has/will your district conduct vaccine clinics for staff, students, and families?* 5. What concerns have you heard within your school community about why people have not gotten vaccinated? 6. What reasons have you heard for why people within your school community have chosen vaccination? 7. With the availability of vaccines, how important will it be for teachers, staff, and students to have access to COVID-19 testing when the new school year starts this fall? |
| **Now we’ll talk a bit about masking and other mitigation measures.**   1. What helps mitigate the perceived risks of COVID-19 in your school?    1. *Probe: testing, vaccination, other assets…* 2. How easy or difficult was it to implement mask mandates in your school? 3. How have other mitigation efforts, such as social distancing, gone in your district? Have those policies changed recently, or do you expect them to change in the near future? |
| **Just a few more questions before we wrap-up.**   1. What are the best ways to share information about this study and testing available through the study with families in your school?    1. *Probes: methods, messengers, frequency…* 2. As a school leader, what were the most important lessons you learned this past year in navigating in-person learning?   Anything else you would like to share? |
