# Supplementary material for: Psychosocial work environment stressors for school staff during the COVID-19 pandemic: Barriers and facilitators for supporting wellbeing
Source: Front Public Health. 2023 Mar 13;11:1096240. doi: 10.3389/fpubh.2023.1096240 (PMC10040557; doi:10.3389/fpubh.2023.1096240)
Supplement: Supplementary file 2 [file Data_Sheet_2.docx]

**T2 Listening Session Guide: District Administrators**

| ***Let’s start with the successes, challenges, and lessons learned that you and your school have experienced related to COVID-19 this past school year.***   1. What 2-3 **strategies and practices** were critical to have in place this school year? Did these strategies and practices change over? How were they different from last school year?    1. *Probes: policies, processes, mitigation strategies -- offering choice of virtual v. at school, classroom pods, PPE for staff* |
| --- |
| 1. What are the top 2-3 things that have worked well in supporting **teachers and staff** with in-person learning? Have those supports changed over time?    1. *Probes: PD, vacation, Pay, child care, etc.* 2. What challenges, if any, have your **teachers and staff** experienced with in-person learning? Have those challenges changed over time?    1. In what ways, if any, have you been able to address these challenges? 3. What are the top 2-3 things that worked well in supporting **students and families** with in-person learning? Have those supports changed over time?    1. What information or other supports did their parents or guardians need?    2. *Probes: hybrid schedule, remote learning, continuing to offer school meals, childcare, and offering laptops, other supports?*      1. What challenges, if any, have your **students and families** experienced related to COVID this school year? Have those challenges changed over time?    1. *In what ways, if any, have you been able to address these challenges?* 2. What resources or information have helped to keep students and staff at school? 3. What resources or information would be helpful to have in continuing to keep students and staff at school? 4. With the emergence of the Omicron variant around the time winter break ended, what factors or reasons influenced how your district, staff, and families chose to respond to this variant or COVID in general?    1. *Probe: any social, behavioral or ethical factors (cultural norms, politics, health, community values)*    2. How did you collect this information from staff and families?    3. What systems and supports do school leaders need to address the Omicron or other future variants?    4. How has that response to COVID-19 and multiple variants changed over this semester and within the past year? 5. Thinking about all that you have just shared, how has your role and responsibilities as a superintendent or assistant superintendent changed as a result of the pandemic?    1. Looking ahead to the next school year, what will your role as superintendent or assistant superintendent look like, if different than today? |
| ***Now let's move on and discuss testing and vaccinations.***   1. What risks related to COVID-19 occurred with in-person learning? How have those risks changed over this semester and within the past year? 2. What role do you see COVID-19 testing having for your school community currently? What about moving forward?    1. *Probe: Why or why not is COVID-19 testing important?* 3. What role, if any, do you think vaccinations play in the spread of the COVID-19 virus? Why is it [insert their response]? 4. To what extent, if any, do schools in your district or your district as a whole promote vaccinations? Are you likely to promote vaccinations to your staff and families in the upcoming months?    1. *Probe: Has/will your district conduct vaccine clinics for staff, students, and families?* 5. What is your school community saying about vaccinations?    1. *Probe: top concerns, reasons why people are getting vaccinated, anything else?* 6. With the availability of vaccines, how important will it be for teachers, staff, and students to have access to COVID-19 testing when the new school year starts this fall? |
| **Now we’ll talk a bit about masking and other mitigation measures.**   1. What helps mitigate the perceived risks of COVID-19 in your school?    1. *Probe: testing, vaccination, other assets…* 2. How have mask mandates gone in your district? What affects (or affected) what students and staff wear a mask? Choose not to wear a mask? Refuse (if required) 3. How have other mitigation efforts, such as social distancing, gone in your district? Have those policies changed recently, or do you expect them to change in the near future? |
| **Just a few more questions before we wrap-up.**   1. What are the best ways to share information about this study and testing available through the study with families in your school?    1. *Probes: methods, messengers, frequency…* 2. As a district leader, what were the most important lessons you learned this past year in navigating in-person learning?   Anything else you would like to share? |
