# Supplementary material for: Psychosocial work environment stressors for school staff during the COVID-19 pandemic: Barriers and facilitators for supporting wellbeing
Source: Front Public Health. 2023 Mar 13;11:1096240. doi: 10.3389/fpubh.2023.1096240 (PMC10040557; doi:10.3389/fpubh.2023.1096240)
Supplement: Supplementary file 3 [file Data_Sheet_3.DOCX]

**T2 Listening Session Guide: Parent & Caregivers**

| 1. So we all know each other, please say your first name, the school your students attend, and what grade that are currently in. For the purposes of today’s session, we are most interested in the experiences of your middle- and high- school students. **[Note: All participants must respond to this question]** |
| --- |
| ***Let’s start with the successes, challenges, and lessons learned that you and your school have experienced related to COVID-19 this past school year.***   1. So far, how has it been learning in person this school year?    1. *Probes: What is the best part(s) about returning to school in person? What has been the hardest part(s) about returning to school in person?* |
| 1. How comfortable or uncomfortable were you with sending your children back into the school building?    1. *Probe: Has your level of comfort changed? Why or why not?* 2. What are the top 2 or 3 things your children’s district and/or school did well that helped you in returning your children to school in person?    1. *Probes: Any concerns (e.g., masking, social distancing, testing, vaccinating)? How does that make you feel?* 3. How easy or hard was it for you to understand the school’s rules and expectations to prevent the spread of COVID-19?    1. *Probes: Which strategies do you think worked the best? Which strategies didn’t work so great?*      1. What steps would the school take if your children were to test positive for COVID?    1. *Probe: To your knowledge, were those steps used for everyone?* 2. Who would you ask or where would you go if you wanted more information on COVID-19?    1. *Probes: In school? Outside of school?* |
| ***Now let's move on and discuss some strategies, including masking, testing, social distancing, and vaccinating.***   1. How important do you think it is to get tested for COVID-19?    1. *Probes: Should everyone at school get tested for COVID when school starts back in the fall? Why or why not? How often?* 2. Tell me, if you or your household members got tested for COVID at your children’s school, what was it like?    1. *Probe: Did this type of access to testing change how comfortable you or your children feel with being at school?* 3. Now, many adults and children can be vaccinated against COVID-19 if they want to. Do you think getting vaccinated should be a requirement for anyone at your children’s school who is eligible?    1. *Probe: Why or why not? Has the increased access to vaccinations changed how comfortable you or your children feel with being at school?* 4. What are your friends, family members, and people in your community saying about the COVID-19 vaccine?    1. *Probe: What is your children’s school district saying about vaccinations?* 5. Did any of your children’s friends from school catch COVID-19?    1. *Probes: How? Did they catch it from someone in or outside school? How did that make you feel?* 6. To your knowledge, did any students come to school even while displaying symptoms of COVID? If so, how did that make you feel?    1. *Probes: Why do you think they still come to school? How easy or difficult would it be for you to quarantine your children if they tested positive for COVID-19? Why?* |
| **Just one more question before we wrap up.**   1. As a parent, what were the most important lessons you learned this past school year about how to support your students?    1. *Probes: Learning/education, school, emotions, COVID, communication, etc.?*   Is there anything else still weighing on your mind about COVID-19 and what it means to return to school that I didn’t ask about? |
