# Supplementary material for: Psychosocial work environment stressors for school staff during the COVID-19 pandemic: Barriers and facilitators for supporting wellbeing
Source: Front Public Health. 2023 Mar 13;11:1096240. doi: 10.3389/fpubh.2023.1096240 (PMC10040557; doi:10.3389/fpubh.2023.1096240)
Supplement: Supplementary file 4 [file Data_Sheet_4.DOCX]

**T2 Listening Session Guide: Staff**

| 1. So we all know each other, please say your first name and the school you work at. **[Note: All participants must respond to this question]** |
| --- |
| ***Let’s start with the successes, challenges, and lessons learned that you and your school have experienced related to COVID-19 this past school year.***   1. So far, how has it been working in person this school year?    1. *Probes: What is the best part(s) about returning to school in person? What has been the hardest part(s) about returning to school in person?* |
| 1. How comfortable or uncomfortable were you with going back into the school building?    1. *Probe: Has your level of comfort changed? Why or why not?* 2. How easy or hard was it for you to understand the school’s rules and expectations to reduce the spread of COVID-19?    1. *Probe: Which strategies do you think worked the best? Which strategies didn’t work so great?* 3. What steps would your school take if you were to test positive for COVID?    1. *Probe: Have these steps changed over time? If yes, how so?*      1. Who would you ask or where would you go if you wanted more information on COVID-19?    1. *Probes: Within the school? Outside of the school?* |
| ***Now let's move on and discuss some strategies, including masking, testing, social distancing, and vaccinating.***   1. What are the top 2 or 3 things your district and/or school did well that helped you in returning to school in person?    1. *Probes: Any concerns (e.g., masking, social distancing, testing, vaccinating)? How does that make you feel?* 2. How important do you think it is to get tested for COVID-19?    1. *Probes: Should everyone at school get tested for COVID when school starts back in the fall? Why or why not? How often?* 3. Tell me, if you or your household members got tested for COVID through the WashU saliva-based testing at your school, what was it like?    1. *Probe: Did this type of access to testing change how comfortable you feel with being at school?* 4. Now, many adults and children can be vaccinated against COVID-19 if they want to. Do you think getting vaccinated should be a requirement for anyone at your school who is eligible?    1. *Probes: Why or why not? Has the increased access to vaccinations changed how comfortable you feel with being at school?* 5. What are your friends, family members, or coworkers saying about the COVID-19 vaccine?    1. *Probe: What is your school district saying about vaccinations? What about students in your classrooms?* 6. To your knowledge, did any students come to school even while displaying symptoms of COVID? If so, how did that make you feel?    1. *Probes: Why do you think they still come to school? How easy or difficult would it be for you to quarantine if you tested positive for COVID-19? Why?* |
| **Just one more question before we wrap up.**   1. As a staff member, what were the most important lessons you learned this past year when returning to in-person learning?    1. *Probes: teaching/education, school, emotions, COVID, communication, etc.?*   Final Thoughts: Is there anything else you would like to share? |
