# Supplementary material for: Psychosocial work environment stressors for school staff during the COVID-19 pandemic: Barriers and facilitators for supporting wellbeing
Source: Front Public Health. 2023 Mar 13;11:1096240. doi: 10.3389/fpubh.2023.1096240 (PMC10040557; doi:10.3389/fpubh.2023.1096240)
Supplement: Supplementary file 5 [file Data_Sheet_5.DOCX]

**T2 Listening Session Guide: Students**

| 1. So we all know each other, please say your first name, the school you attend, what grade you’re currently in, and if you’re attending school in person or virtually. **[Note: All participants must respond to this question]** |
| --- |
| ***Let’s start with the successes, challenges, and lessons learned that you and your school have experienced related to COVID-19 this past school year.***   1. So far, how has it been learning in person this school year?    1. *Probes: What is the best part(s) about returning to school in person? What has been the hardest part(s) about returning to school in person?* 2. COVID-19 has changed many things about our school experience. How easy or hard was it for you to understand your school’s rules and expectations to prevent the spread of COVID-19?    1. *Probes: Which strategies do you think worked the best? Which strategies didn’t work so great?* 3. What steps would your school take if you were to test positive for COVID?    1. *Probe: Have these steps changed over time? If yes, how so?* 4. Who would you ask or where would you go if you wanted more information on COVID-19?    1. *Probes: In school? Outside of school?* |
| ***Now let's move on and discuss some strategies, including masking, testing, social distancing, and vaccinating.***   1. What are the top 2-3 things your school did well that helped you in returning to school in person?    1. *Probes: Any concerns (e.g., masking, social distancing, testing, vaccinating)? How does that make you feel?* 2. How important is getting tested for COVID-19?    1. *Probes: Should everyone at school get tested for COVID when school starts back in the fall? Why or why not? How often?* 3. Tell me, if you got tested for COVID at your school through the WashU saliva-based testing, what was it like?    1. *Probe: Did it change how comfortable you were with being in-person at school?*      1. Now, many adults and children can be vaccinated against COVID-19 if they want to. Do you think your school should make getting vaccinated a requirement for anyone at your school who is eligible?    1. *Probe: Why or why not?* 2. What are your friends and families saying about the COVID-19 vaccine?    1. *Probe: What is your school district saying about vaccinations?* 3. Did any of your friends from school catch COVID-19?    1. *Probes: How? Did they catch it from someone in or outside school? How did that make you feel?* 4. How does it make you feel whenever students come to school even though they display COVID-like symptoms?    1. *Probes: Why do you think they still come to school? How comfortable would you feel telling a teacher, nurse, or other staff member if you felt sick at school?* |
| **Just a few more questions before we wrap up.**   1. As a student, what were the most important lessons you learned this past year when returning to in-person learning?    1. *Probes: Learning/education, school, emotions, COVID, communication, etc.?*   That’s all the questions I have for you today. Is there anything else still weighing on your mind about COVID-19 and what it means to return to school that I didn’t ask about? |
